# Editorial: Pathogenic aspects of the innate immune system of the kidney

**DOI:** 10.3389/fmed.2024.1360450

**Published:** 2024-01-29

**Authors:** Takashi Oda, Rui Zeng, Hiroyuki Nakashima

**Affiliations:** ^1^Department of Nephrology and Blood Purification, Kidney Disease Center, Tokyo Medical University Hachioji Medical Center, Hachioji, Tokyo, Japan; ^2^Tongji Medical College, Tongji Hospital, Huazhong University of Science and Technology, Wuhan, China; ^3^Department of Immunology and Microbiology, National Defense Medical College, Tokorozawa, Saitama, Japan

**Keywords:** innate immune system, monocyte/macrophages, polymorphonuclear leukocytes, NK/NKT cells, acute kidney injury (AKI), chronic kidney disease (CKD)

The innate immune system serves as an important biological defense system against various pathogens within hosts. However, under certain conditions, it can pose a pathogenic threat to the host itself. The kidney is one of the targets of such attack by components of the innate immune system. Several components, including monocyte/macrophages (Mφ), polymorphonuclear leukocytes, and NK/NKT cells, have been implicated in the development of both acute kidney injury (AKI) (1) and chronic kidney diseases (CKD) of various causes, such as diabetic kidney disease (DKD) (2), acute/chronic glomerulonephritis, tubulointerstitial nephritis, renal vasculitis (3), thrombotic microangiopathy (TMA), and kidney transplant rejection (4).

Recent advances in the field of molecular targeted therapy have facilitated the development of novel therapeutic options tailored to the specific pathogenic targets of diseases. Considering the nature of the innate immune system, its role would be in the early phase of the disease process. Recognizing that targeting the earlier phases of a disease is more effective than later disease stages, understanding the pathogenic aspects of the innate immune system as a therapeutic target for kidney injury becomes paramount.

In this Research Topic entitled “Pathogenic Aspects of the Innate Immune System of the Kidney,” we published three original research articles and one review article, all of which focus on the roles of innate immune cells in various kidney injuries.

First, all three original articles are related to the roles of Mφ in various renal diseases. Mφ is one of the major phagocytic cell components of innate immune system with versatile functions (5). It is divided into tissue-resident Mφ (F4/80^high^CD11b^low^ in mice) and infiltrating Mφ (F4/80^low^CD11b^high^ in mice), and pro-inflammatory M1 Mφ and anti-inflammatory/tissue repair M2 Mφ. Roles of various Mφ in the damage to the kidney are summarized in Figure 1.

**Figure 1:**
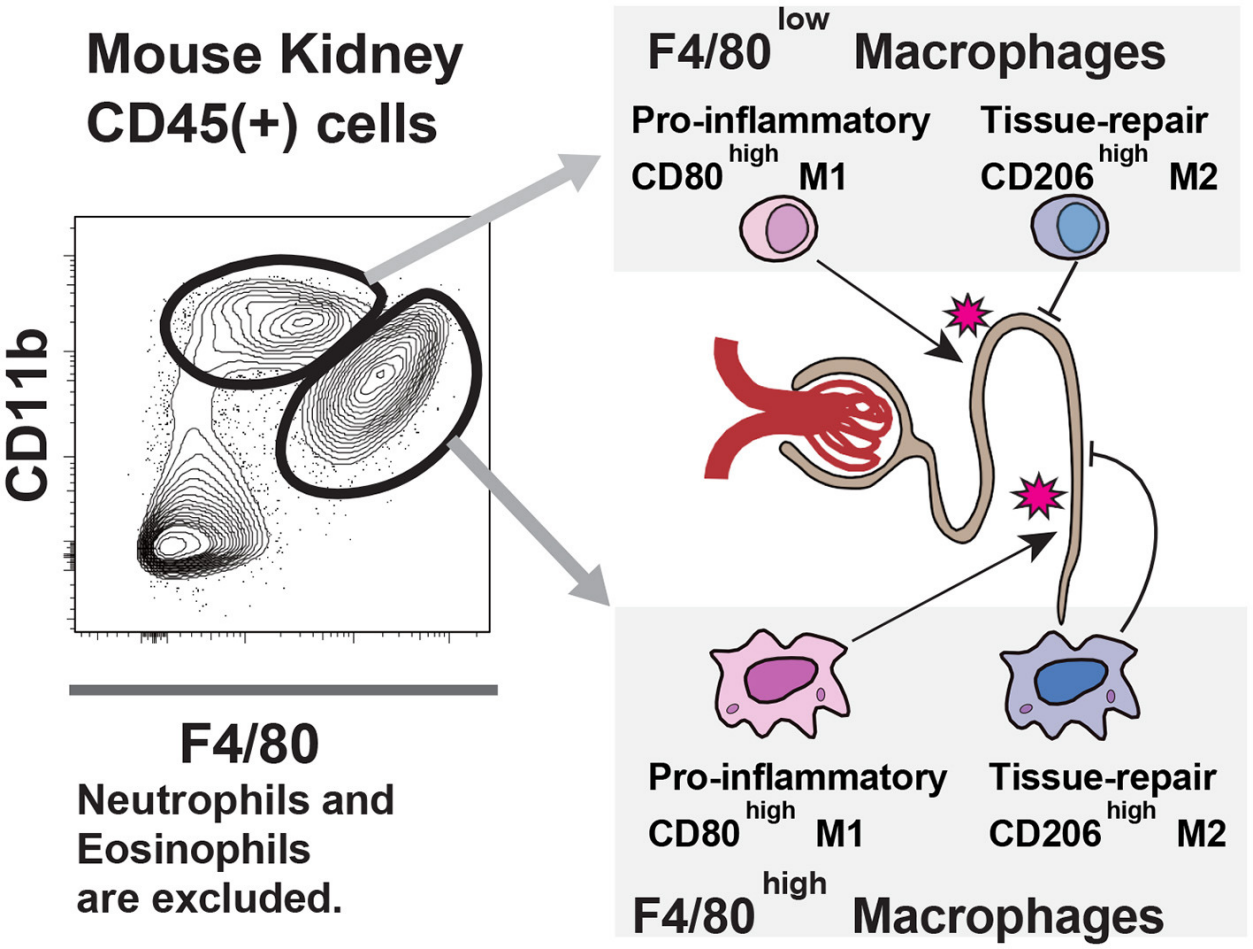
Macrophage populations in the kidney: In the kidney, two macrophage populations exist; one is F4/80 high, and the other is low. Like other organ's macrophage populations, such as liver Kupffer cells and pulmonary alveolar macrophages, F4/80 high cells are tissue-resident, and F4/80 low cells are monocyte-derived populations. Both macrophages express M1 marker CD80 and M2 marker CD206. The balance between these antigens defines their function, i.e., proinflammatory or tissue repair. The dynamics of these macrophage populations in experimental models are crucial for understanding the pathological mechanisms.

Based on the previous findings on the association of the coagulation process with organ fibrosis (6), Oh et al. evaluated the expression of coagulation factors on Mφ in renal tissues with ischemia–reperfusion injury at acute (AKI) and chronic phases (CKD, fibrosis) in mice. Interestingly, they found increased production of key coagulation factors both by infiltrating and resident renal Mφ, suggesting the novel mechanism of renal fibrosis through fibrinogenesis induced by upregulated production of coagulation factors by renal Mφ and subsequent matrix deposition. Thus, anti-coagulation therapy might be the therapeutic option for renal fibrosis.

It is well known that AKI is more severe in the elderly and has a higher rate of transition to CKD (7, 8). Furthermore, age-related changes in the gut environment have reportedly been associated with age-related diseases through the exacerbation of chronic inflammation (9). Therefore, Kim et al. compared renal and gut histology in aged and young mice with bilateral renal ischemia–reperfusion injury. Their experiment revealed that AKI in aged mice induced gut dysbiosis, which prolonged intestinal and renal inflammation with immune cell infiltration such as Mφ, neutrophils, and Th17 cells, leading to additional fibrosis progression in the kidney. Based on these results, they suggested that the gut–kidney axis may be an important mechanism of AKI exacerbation in the elderly and may be a novel therapeutic target for aging-related renal disease.

Meanwhile, Sadaka et al. analyzed the exacerbation mechanism of cystic growth in ADPKD using a mouse model with a conditional genetic deletion of *pkd1* (10). In this model, they observed accelerated cystogenesis in response to chronic dietary protein overload, consistent with a previous finding (11). Through precise histological analysis of this model subjected to a high protein diet, they identified increased glutamine delivery and alternative energy production during the early disease phase, without Mφ infiltration, with inflammation with Mφ infiltration developing in the later disease phase, resulting in accelerated cystogenesis. The authors confirmed involvement of this mechanism by showing that accelerated cyst growth induced by chronic high protein diet could be attenuated by liposomal clodronate-mediated Mφ depletion in this model.

In the only review article within this Research Topic, Goto et al. explore the roles of innate immune system cells in heatstroke-induced AKI (12, 13). Heat stress can induce renal tubular damage directly or indirectly through inflammatory immune responses leading to AKI. Recent observations have revealed that heatstroke-induced AKI is not a temporary condition but progresses to CKD (14, 15). Therefore, the authors comprehensively summarized the important roles of cell components of the innate immune system, such as neutrophils, Mφ, lymphocytes (NK, NKT cells, cytotoxic CD8^+^ cells), and mast cells on disease process of heatstroke-induced AKI and AKI to CKD transition. Further studies are required to uncover the complex interactions among these various innate immune cells in each disease process.

Knowledge in this area is expanding, and continued advancement is expected.

## Author contributions

TO: Writing – original draft, Writing – review & editing. RZ: Validation, Writing – review & editing. HN: Validation, Visualization, Writing – review & editing.

## References

[1] Uchida T Nakashima H Ito S Ishikiriyama T Nakashima M Seki S. Activated natural killer T cells in mice induce acute kidney injury with hematuria through possibly common mechanisms shared by human CD56+ T cells. Am J Physiol Renal Physiol. (2018) 315:F618–27. 10.1152/ajprenal.00160.2018 29993279 PMC6172573

[2] Ito S Nakashima H Ishikiriyama T Nakashima M Yamagata A Imakiire T. Effects of a CCR2 antagonist on macrophages and toll-like receptor 9 expression in a mouse model of diabetic nephropathy. Am J Physiol Renal Physiol. (2021) 321:F757–70. 10.1152/ajprenal.00191.2021 34719947

[3] Nakazawa D Marschner JA Platen L Anders HJ. Extracellular traps in kidney disease. Kidney Int. (2018) 94:1087–98. 10.1016/j.kint.2018.08.035 30466565

[4] Koenig A Mezaache S Callemeyn J Barba T Mathias V Sicard A. Missing self-induced activation of NK cells combines with non-complement-fixing donor-specific antibodies to accelerate kidney transplant loss in chronic antibody-mediated rejection. J Am Soc Nephrol. (2021) 32:479–94. 10.1681/ASN.2020040433 33239394 PMC8054908

[5] Tang PM Nikolic-Paterson DJ Lan HY. Macrophages: versatile players in renal inflammation and fibrosis. Nat Rev Nephrol. (2019) 15:144–58. 10.1038/s41581-019-0110-2 30692665

[6] Oh H Park HE Song MS Kim H Baek JH. The therapeutic potential of anticoagulation in organ fibrosis. Front Med (Lausanne). (2022) 9:866746. 10.3389/fmed.2022.866746 35652066 PMC9148959

[7] Coca SG. Acute kidney injury in elderly persons. Am J Kidney Dis. (2010) 56:122–31. 10.1053/j.ajkd.2009.12.034 20346560 PMC2902696

[8] Schmitt R Coca S Kanbay M Tinetti ME Cantley LG Parikh CR. Recovery of kidney function after acute kidney injury in the elderly: a systematic review and metaanalysis. Am J Kidney Dis. (2008) 52:262–71. 10.1053/j.ajkd.2008.03.005 18511164

[9] Fransen F van Beek AA Borghuis T Aidy SE Hugenholtz F van der Gaast-de JC. Aged gut microbiota contributes to Systemical Inflammaging after transfer to germ-free mice. Front Immunol. (2017) 8:1385. 10.3389/fimmu.2017.01385 29163474 PMC5674680

[10] Piontek KB Huso DL Grinberg A Liu L Bedja D Zhao H. A functional floxed allele of Pkd1 that can be conditionally inactivated *in vivo*. J Am Soc Nephrol. (2004) 15:3035–43. 10.1097/01.ASN.0000144204.01352.86 15579506

[11] Warner G Hein KZ Nin V Edwards M Chini CC Hopp K. Food restriction ameliorates the development of polycystic kidney disease. J Am Soc Nephrol. (2016) 27:1437–47. 10.1681/ASN.2015020132 26538633 PMC4849816

[12] Sorensen C Hess J. Treatment and prevention of heat-related illness. N Engl J Med. (2022) 387:1404–13. 10.1056/NEJMcp2210623 36170473

[13] Epstein Y Yanovich R. Heatstroke. N Engl J Med. (2019) 380:2449–59. 10.1056/NEJMra1810762 31216400

[14] Kupferman J Ramírez-Rubio O Amador JJ López-Pilarte D Wilker EH Laws RL. Acute kidney injury in sugarcane workers at risk for mesoamerican nephropathy. Am J Kidney Dis. (2018) 72:475–82. 10.1053/j.ajkd.2018.04.014 30042041

[15] Tseng MF Chou CL Chung CH Chen YK Chien WC Feng CH. Risk of chronic kidney disease in patients with heat injury: a nationwide longitudinal cohort study in Taiwan. PLoS ONE. (2020) 15:e0235607. 10.1371/journal.pone.0235607 32614909 PMC7332078

